# Long Non-Coding RNA PVT1 and Its Target miRNA-146a as Potential Prognostic Biomarkers in Rheumatoid Arthritis Patients

**DOI:** 10.3390/life11121382

**Published:** 2021-12-10

**Authors:** Randa Erfan, Olfat G. Shaker, Mahmoud A. F. Khalil, Yumn A. Elsabagh, Azza M. Ahmed, Abeer K. Abu-El-Azayem, Mohamed S. Gomaa, Asmaa Mohammed

**Affiliations:** 1 Department of Biochemistry and Molecular Biology, Faculty of Medicine, Cairo University, Cairo 11956, Egypt; randa.erfan@cu.edu.eg (R.E.); Olfat.shaker@kasralainy.edu.eg (O.G.S.); 2 Department of Microbiology and Immunology, Faculty of Pharmacy, Fayoum University, Fayoum 63514, Egypt; 3 Department of Internal Medicine, Rheumatology and Immunology Unit, Cairo 11956, Egypt; Dr_youm@yahoo.com; 4 Department of Rheumatology, Faculty of Medicine, Fayoum University, Fayoum 63514, Egypt; ama56@fayoum.edu.eg; 5 Department of Medical Microbiology and Immunology, Microbiology, Faculty of Medicine, Cairo University, Cairo 11956, Egypt; abeer.aboualazaim@kasralainy.edu.eg; 6 Department of General Medicine, Faculty of Medicine, Fayoum University, Fayoum 63514, Egypt; mss04@fayoum.edu.eg; 7 Department of Biochemistry and Molecular Biology, Faculty of Medicine, Fayoum University, Fayoum 63514, Egypt; amm18@fayoum.edu.eg

**Keywords:** lncRNAs, miRANs, lnc-PVT1, miR-146a, rheumatoid arthritis, osteoarthritis

## Abstract

Objective: Long non-coding RNAs (lncRNAs) and their target microRNAs were documented in multiple studies to have a significant role in different joint disorders such as rheumatoid arthritis (RA) and osteoarthritis (OA). The current work aimed to determine the potential role of lnc-PVT1 and miR-146a as promising biomarkers to distinguish between RA, OA patients, and healthy individuals. Methods: The expression levels of lnc-PVT1 and its target miR-146a in the serum were measured for three different groups, including patients with RA (40), OA patients (40), and healthy controls (HCs) (40). Participating individuals were subjected to a full history investigation and clinical examination. Blood samples were tested for ESR, RF, CBC, as well as liver and renal functions. Serum was used to detect the relative expression levels of lnc-PVT1 and miR-146a and we correlated the levels with RA and OA activity and severity signs. Results: Lnc-PVT1 expression level was greater among patients with RA compared to that of OA patients, with a fold change median of 2.62 and 0.22, respectively (*p* = 0.001). The miR-146a fold change was significantly demonstrated between the RA, OA, and HCs groups. There was no correlation between both biomarkers with the disease activity scales (DAS28) of RA, the Knee injury Osteoarthritis Outcome Score (KOOS), or any sign of detection of the disease severity of OA. Conclusions: lnc-PVT1 and miR-146a could be considered as promising biomarkers for the diagnosis of RA and OA and may have an important role as therapeutic targets in the future.

## 1. Introduction

Rheumatoid arthritis (RA) is a common chronic autoimmune disease of the joints. RA is characterized by cartilage destruction, chronic inflammation of joints (mainly those of the hands and feet), positive rheumatoid factor (serum), as well as abnormal synovial hyperplasia, which subsequently causes joint deformities and dysfunction [1,2,3]. Currently, the exact cause of RA remains unknown but it is usually associated with other systemic pathologies [2]. Although not fully understood, the pathogenesis and development of RA may be directly correlated with the anatomical and physiological structure of the joint involved, as well as the chronic synovial inflammation and bone erosion due to the activated fibroblast-like synoviocyte (FLSs) [4,5]. The development of RA involves; (1) FLSs proliferation, (2) lymphocytic infiltration/invasion, (3) neovascular formation which allows for the invasion of the cartilage surface by the synovial membrane to form a pannus, and (4) subsequent destruction of both the cartilage and the bone that causes structural and functional abnormalities [6].

Osteoarthritis (OA) is a prevalent musculoskeletal disease found in about 15% of the population [1]. In contrast to RA, the pathogenesis of OA involves the erosion of bone and permanent annihilation of the articular cartilage, which is activated by the stimulation of pro-inflammatory cytokines (TNF-α, IL-1, and IL-6). Cytokines promote the production of metalloproteinase (MMP) and collagenase, which in turn, degrade type II collagen (via collagenase 1 and 3, a biochemical hallmark of OA) [7], as well as inhibit the production of collagen, collagenase inhibitors, and proteoglycans [8]. Risk factors of OA include elderly, gender, individuals with a high BMI, ethnic background, trauma, as well as any occupation that may imply stress on joints (e.g., pressure and weight-bearing activities) [9]. Unfortunately, the standard medication used in the treatment of various arthropathies is frequently associated with serious adversities [10] including hemorrhage, NSAID-induced nephrotoxicity, and gastrointestinal ulcers [11]. Regarding the majority of RA patients, biological therapies are highly effective. Until now, the pathogenesis of RA is not fully known, but some clinical studies have found that long non-coding RNAs (lncRNAs), which are non-protein-coding RNAs (>200 nucleotides in length), may be involved [12,13].

LncRNAs can regulate the expression of the substances they target via epigenetics, alternate splicing, transcriptional/translational regulation, as well as small RNA sponging [14]. They are required in various inflammatory pathologies [15,16]. For example, quercetin stimulates FLS apoptosis via increasing MALAT1 in RA [17]. In OA, elevated levels of lnc-PVT1 stimulate chondrocyte apoptosis [18], while in RA, lnc-PVT1 knockdown decreases the inflammatory response and activates FLS apoptosis [19]. LncRNAs exert their biological actions via sponging the targeted miRNAs (20–22 nucleotides in length) [20], which are responsible for the regulation of target mRNAs via sequence complementarity [21]. Although some studies have found that an increased number of miRNAs may be linked to the pathogenesis of RA [22,23], the exact mechanism requires extensive investigation. It has been suggested that miR-146a is an essential component of both the innate and adaptive immune system (expression profile of its cellular components) as well as autoimmune disorders including sjogren’s syndrome (SS), systemic lupus erythematosus (SLE), multiple sclerosis (MS), and RA. Patients with lupus demonstrated reduced levels of miR-146a within the peripheral blood mononuclear cells (PBMC), whereas patients with RA presented with elevated levels of miR-146a within the PBMC.

MicroRNA-146a (miR-146a), lnc-PVT1 target, is considered a biomarker and indicator of chronic respiratory illnesses, especially those of an obstructive nature [24]. It can also be used to differentiate between patients with stable chronic obstructive pulmonary disorders (COPD) and those with acute COPD (AECOPD) [24]. The interaction between lnc-PVT1 and miR-146a not only helps with the management of the disease but is also used to monitor the risk of developing any acute exacerbation of COPD [25]. Wang and co-workers developed an RA rat model to evaluate the expression of lnc-PVT1, miR-543, and signal peptide-CUB-EGF-like containing protein 2 (SCUBE2) in synovial tissues. According to Wang’s findings, lnc-PVT1 knockdown has the potential to limit RA progression by suppressing SCUBE2 expression to sponge miR-543 [26]. Furthermore, few studies have suggested that lnc-PVT1 may play a role in the development and progression of RA [20,27]. To the best of our knowledge, this is the first report designed to determine the connection between lnc-PVT1 and its target miR-146a in human to understand its impact on the clinical course and disease development in patients with RA and OA.

## 2. Materials and Methods

The local ethics committee of Fayoum University Hospital approved the current study protocol (Code: R183). Accordingly, all procedures followed the standards of the Declaration of Helsinki.

### 2.1. Study Design and Patients

This study included 80 patients (40 with RA and a similar number of OA patients) and 40 healthy volunteers. Patients were selected from the Department of Rheumatology at Fayoum University Hospital, Egypt. The control group consisted of 40 volunteers who had no family history of RA or any autoimmune disorder. Before being enrolled, all patients were asked to fill out an informed consent form. Any patient with an autoimmune disease, cancer, a chronic infectious disease, or a new infection within a month of enrollment was excluded.

### 2.2. Rheumatoid Arthritis Patients

The study included 40 patients with RA according to the 2010 American College of Rheumatology (ACR)/European League against Rheumatism (EULAR) criteria [28]. All patients have undergone a complete history investigation, clinical examination, assessment of the tender and swollen joint count (SJC/TJC), as well as assessment of the disease activity score (DAS-28) [29]. Any assessment in the form of a questionnaire was given in an Arabic version. The health assessment questionnaire was calculated (ranging from 0 to 2) [30]. Laboratory investigations included liver function tests, renal function tests, rheumatoid factor, as well as erythrocyte sedimentation rate (ESR). Regarding radiographic evaluation, antero-posterior plain radiographs of both hands and wrists were done to determine the joint space narrowing (JSN), erosion, and osteopenia as illustrated in the schematic outline (Figure S1a).

### 2.3. Osteoarthritis Group

OA patients were diagnosed using the ACR criteria [31] supported by radiographic evidence. Patients excluded from the study were those that presented with any sign of secondary OA, muscle strain involving the lower extremities, neurological disorders, inflammatory arthritis, and ligament sprain. Radiographic imaging was used to assess the degree of involvement, including the presence of JSN, the formation of a subchondral cyst, and any marginal spur formation. A blinded (from the study) radiologist evaluated the results of imaging using the Kellgren and Lawrence (KL) grading, which has high intra-rater reliability [32]. This grading ranges from a scale of (0) to (4), where (0) refers to no radiological indications of OA, (1) indicates doubtful osteophyte, (2) indicates definite osteophyte, (3) indicates the presence of moderate JSN, and (4) indicates severely narrowed joint spacing. The Knee injury and Osteoarthritis Outcome Score (KOOS) Arabic version was used [33]. For detecting disease severity, 6 MWT [34], second Chair Stand Test, and Stair Climb test were performed [35] as illustrated in the schematic outline (Figure S1b).

### 2.4. Data Collection and Laboratory Investigation

At registration, the following data was collected from patients and controls: age, gender, clinical presentation, and BMI. The blood samples were collected for measurement of urea, creatinine, ALT, AST, ESR, HB, WBCs, and PLT. Relative expressions of the two biomarkers were studied: lnc-PVT1 and miR-146a. The vacutainer device was used to collect (5 mL) blood from each subject. Tubes, which had separator gels lodged between the serum layer (top) and the packed cells, contained the collected blood samples and were left to clot for a total of 15 min before being centrifuged at 4000× *g* for 10 min. After isolation from the clotted whole blood, the serum was stored at −80 °C until required.

### 2.5. Extraction of RNA

The expression of lncRNAs was evaluated via real-time PCR. With the QIAzol lysis reagent, RNA was extracted using the miRNeasy extraction kit (Qiagen, Valencia, CA, USA). The RNA concentration was determined via the NanoDrop2000 (Thermo Scientific, Wilmington, NC, USA). The extracted RNA was then stored at a temperature of −80 °C.

### 2.6. LncRNAs Expression Using Quantitative RT-PCR

Sixty nanograms (ng) of RNA were used during the reverse transcription (RT). RT2 first strand kit (Qiagen, Santa Clarita, CA, USA) was used. The final volume of the RT reaction was 20 µL, according to the manufacturer’s instructions. Gene expression of PVT1 was normalized to GADPH expression while gene expression of miR-146a was normalized to SNORD68 (Cat No MS00033712) expression. Forwarded (5′-TGAGAACTGTCCTTACGTGACC-3′), and reverse (5′-AGAGCACCAAGACTGGCTCT-3′) was the lnc-PVT1 primer. (MiR-146a) sequence (Qiagen, Valencia, CA, USA) (Cat No MS00003535). The primer sequences for GAPDH were as follows: F 5′-CCCTTCATTGACCTCAACTA-3′, R 5′-TGGAAGATGGTGATGGGATT-3′.

RT-PCR was conducted using 20 µL reaction mixtures, which were composed of a 10 µL master mix, 2.5 µL of diluted cDNA, 1 µL readymade assay primer, and 5.5 µL RNAase-free water by Rotor-Gene Q System. The conditions of the PCR were as follows: initially at 95 °C for 10 min, followed by a total of 45 cycles at 95 °C for 15 s, and finally 60 °C for 60 s. Relative to the internal control (2^−∆Ct^), the gene expression was calculated. To determine the specificity of the RT-PCR reactions, a melt curve analysis was performed. Using 2^−∆∆Ct^ for relative quantification, the fold change was determined.

### 2.7. Study’s Outcomes

The main parameter was fold change in the two studied lncRNAs, lnc-PVT1 and miR146a, and their diagnostic utilities in RA and OA patients. The secondary outcomes included the association between the fold changes in the studied biomarkers and the clinical activity of the diseases, clinical presentation, and treatment modality.

### 2.8. Statistical Analysis

All data was gathered and analyzed using the Statistical Package of Social Science (SPSS) software version (22) (SPSS Inc., Chicago, IL, USA). For qualitative data, the Chi-square test (for two or more groups) was used, and the data were presented in the form of numbers and percentages. An independent *t*-test (for two independent groups) and one-way ANOVA (for two or more independent groups) were used for quantitative information, which was presented in the form of standard deviation. Quantitative data included in this study was first tested for normality by the One-Sample Kolmogorov–Smirnov test in each study group, and then inferential statistical tests were selected. A bivariate Pearson correlation test was used to determine the association between variables. The specificity and sensitivity of the studied variable were evaluated using a ROC curve analysis. Statistical significance was considered if *p*-values were less than 0.05.

## 3. Results

The studied subjects were categorized into three groups: RA patients, OA patients, and HCs subjects. Each group is composed of 40 individuals. The mean age was 38.4 ± 10.1, 53.5 ± 6.7, and 52.8 ± 8.4 for the RA, OA, and HCs groups, respectively. The mean BMI for each group was 30.4 ± 5.3 kg/m^2^, 31.04 ± 4.7 kg/m^2^, 27.8 ± 5.1 kg/m^2^, respectively. The percentage of females in each group was 87.5% (RA), 75% (OA), and 82.5% (controls) (Table 1). The laboratory investigations of different groups are illustrated in Table 2.

### 3.1. Expression Levels of lnc-PVT1 and miR-146a among Cases

**Figure 1:**
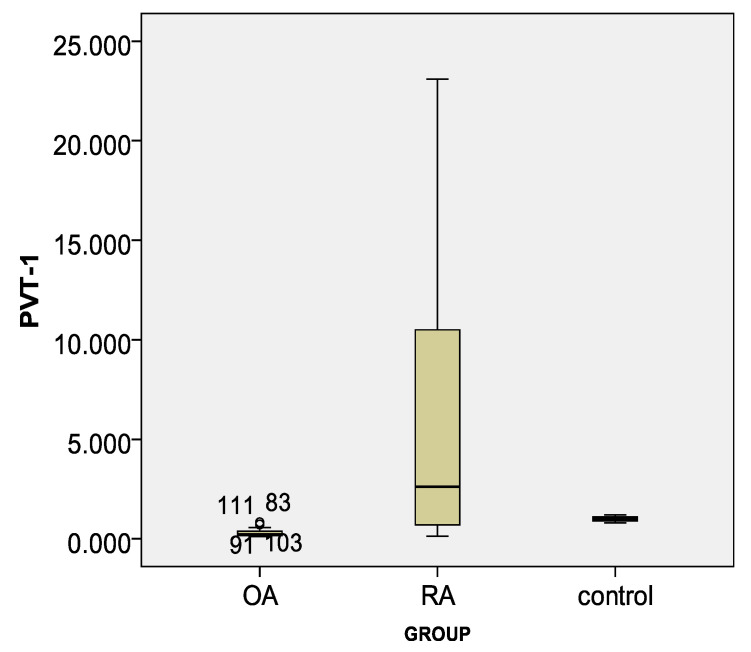
Box plot for lnc-PVT1 marker in different study groups.

**Figure 2:**
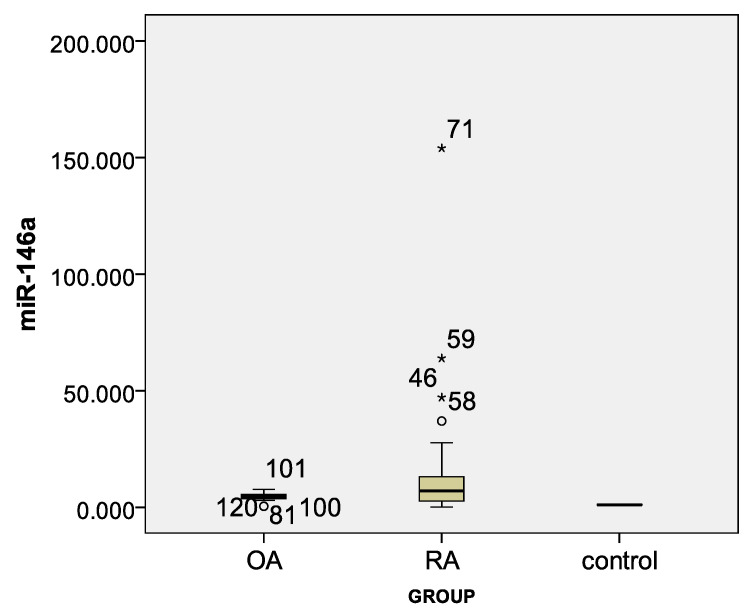
Box plot for miR-146a marker in different study groups. (*) refers to odds values.

Lnc-PVT1 was elevated in patients with RA compared to the OA group, with a fold change median 2.62 and 0.22, respectively (*p* = 0.001), and compared to HCs with an increase of 2 fold (Table 3). Overexpressed miR-146a marker fold change was demonstrated between both the RA and OA groups and controls but no variation between the RA and OA groups (Table 3, Figure 1 and Figure 2).

### 3.2. Correlation between lnc-PVT1 and miR-146a Markers with Disease Activity among RA Group

All patients were subjected to assessment of (DAS-28), (SJC), (ESR), and rheumatoid factor. As regards imaging, antero-posterior plain radiographs of both hands and wrists were done to evaluate the level of joint space narrowing (JSN), erosion, and osteopenia (Table 4). A positive correlation was found between the lnc-PVT1 marker and SJC among the RA group. However, there was no association between miR-146a and other variables (Table 5).

### 3.3. Sensitivity and Specificity of lnc-PVT1 and miR-146a in RA Diagnosis

**Figure 3:**
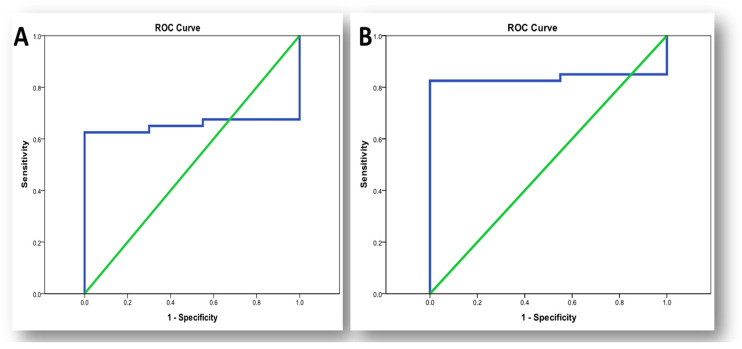
ROC curve for biomarkers in diagnosis of RA. (**A**) ROC curve for lnc-PVT1 in diagnosis of RA; (**B**) ROC curve for miR-146a in diagnosis of RA.

Lnc-PVT1 had a sensitivity of 62.5% and specificity of 90% at cut off 1.15 (AUC = 65.4%, 95% CI: 0.51–0.79). The sensitivity and specificity of MiR-146a were 82.5% and 100%, respectively, with a cut off of 1.433 (AUC = 83.6%, 95% CI: 0.72–0.95) (Table 6, Figure 3).

### 3.4. Correlation between PVT1 and miR146a Markers with Demographic and Laboratory Investigations among RA Group

There was no statistically significant correlation (*p*-value > 0.05) between PVT1 and miR-146a markers level with age, BMI, and all laboratory investigations among the RA group (Table 7).

### 3.5. Correlation between lnc-PVT1 and miR-146a Markers with Disease Severity Scales among OA Group

The mean total KOOS score was 47.3 ± 13.4, for the chair stand test. It was 11.4 ± 6.9, and 19.7 ± 8 for the mean stair climb test. Furthermore, 37.5% of the OA group had stage 2 Kellgrn–Lawrence Grading radiological Scale, 42.5% had stage 3, and 20% had stage 4 (Table 8). No significant correlation was found between lnc-PVT1 and miR-146a markers with different disease severity scales.

### 3.6. Sensitivity and Specificity of lnc-PVT1 and miR-146a in OA Diagnosis:

**Figure 4:**
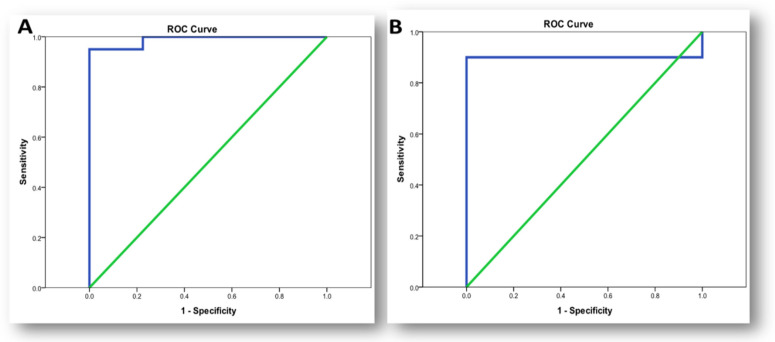
ROC curve for biomarkers in diagnosis of osteoarthritis. (**A**) ROC curve for lnc-PVT1 in diagnosis of OA; (**B**) ROC curve of miR-146a in diagnosis of osteoarthritis.

Sensitivity and specificity test for lnc-PVT1 showed a good predictive power in diagnosis of OA with sensitivity of 100% and specificity of 77.5% at cut off (0.865) (AUC = 98.9%, 95% CI: 0–1) versus 90% and 100%, respectively, for miR-146a at cut off (2.05) (AUC = 90%, 95% CI: 0.81–0.99) (Table 9, Figure 4).

### 3.7. Correlation between PVT1 and miR146a Markers with Demographic and Laboratory Investigations among OA Group

There was no statistically significant correlation (*p*-value > 0.05) between PVT1 and miR-146a markers level with age, BMI, and all laboratory investigations among OA group (Table 10).

## 4. Discussion

Several studies examined the expression of lncRNAs in RA patients and their regulatory role in inflammation and oxidative stress of synovial fibroblasts [36,37,38,39]. Many reports have revealed interactions between lncRNA and miRNA, as well as their conurbations, in the pathogenesis of rheumatoid arthritis (RA) [27,36,40,41,42,43]. In addition, the biological significance of lncRNA in OA, as well as the mechanisms that underpin it, has also been extensively studied [44,45,46,47]. For the first time, we studied the relationship between the expression of lnc-PVT1 and its target miRNA (miR-146a) and the pathogenesis of RA compared to OA. Lnc-PVT1 located at 8q24.21, is a novel biomarker for diagnosing cancer. It is very easy to measure its level in the serum as well as the saliva and is characterized by high specificity [48]. Moreover, lnc-PVT1 exerts regulatory effects via miRNAs modulation (cytoplasmic level), on gene transcription and protein synthesis [49]. Its role in inflammatory responses has recently been discovered [20,50]. As demonstrated in septic rats, lnc-PVT1 is highly expressed in myocardial tissues. Lnc-PVT1 reduces cardiac function and modulates the levels of inflammatory cytokines via activating mitogen-activated protein kinase (MAPK)/nuclear factor (NF)-κB pathway [51], which may suggest that it is an important contributing factor in various inflammatory diseases.

To better understand the role of lncPVT1 in the pathogenesis of RA and its susceptibility, we examined lncPVT1 expression in RA patients, OA patients, and healthy controls (HCs) and used ROC curves to assess its ability to distinguish between them. We found that lnc-PVT1 expression levels were significantly higher in RA patients, than in OA patients or HCs. Moreover, lnc-PVT1 expression levels are not correlated to disease activity, so this lncRNA may be related to the pathogenesis of RA. More importantly, ROC analysis reported that the level of lnc-PVT1 in the serum of RA patients can distinguish RA patients from healthy controls with an AUC of 0.654 at a cut off value of ≥1.15 folds with a sensitivity and specificity of 62.5% and 90%, respectively. Likewise, Zhang et al., detected elevated lnc-PVT1 expression and decreased *sirt6* expression within the synovial tissues of RA-FLSs rat models. Lnc-PVT1, commonly located within the nucleus, can bind to the *sirt6* promoter in order to stimulate *sirt6* methylation, therefore inhibiting the transcription of *sirt6*. Lnc-PVT1 knockdown restored the expression of *sirt6* via reducing the methylation of *sirt6*, thereby alleviating RA [20]. Its knockdown reduced the proliferation of cells and stimulated cellular apoptosis in RA-FLSs via targeting miR-145–5p [27]. Lnc-PVT1 may stimulate inflammatory responses by activation of the MAPK/NF-κB pathway or by sponging specifically targeted miRNAs, thereby facilitating the synthesis and release of inflammatory cytokines [52]. According to Tang et al., TNF-induced RA-FLS over-proliferation was suppressed by knocking down PVT1, while TNF-induced RA-FLS apoptosis was reversed. TNF-induced production of interleukin (IL)1 and IL6, as well as NF-κB activation through miR-145-5p, were also suppressed after PVT1 knockdown [27].

MiR-146a is needed for modulating the function and differentiation of both innate and adaptive immune cells. It (or its SNP) has been confirmed as a target miRNA of lnc-PVT1 in multiple diseases (prostate cancer, colon cancer) [53]. Based on this information, we hypothesized that there may be a correlation between miR-146a and lnc-PVT1, and therefore this would be associated with the progression of RA. Interestingly, our results determined that miR-146a was up-regulated in both RA and OA patients when compared to the controls, but it neither correlated with both diseases severity signs nor with lnc-PVT1 expression levels. However, Churov et al. documented that the specificity and sensitivity of a single miRNA as a biomarker of RA is generally weak, and it is better to depend on a set of several miRNAs or a mixture of miRNAs and other parameters to demonstrate an effective diagnostic tool [54]. Zhang and coworkers found that miR-146a exacerbated pro-inflammatory cytokines by decreasing cartilage matrix-associated gene expression. MiR-146a modulates cartilage homeostasis by targeting calcium/calmodulin-dependent protein kinase II delta (Camk2d) and protein phosphatase 3, regulatory subunit B, beta isoform (Ppp3r2, also known as calcineurin B, type II). Their findings indicate that miR-146a plays a vital role in cartilage homeostasis [53]. Interestingly, MiR-146a expression was shown to be higher in CD4+ T cells, and it was found to be positively linked with TNF and IL-17 levels. The increased expression of miR-146a may contribute to RA inflammatory pathways by reducing T-cell death and increasing IL-17 cell development, respectively. Additionally, the expression of miR-146a in CD4+ T cells is inversely correlated with Fas-associated factor 1 (FAS-1), which controls T-cell death [54].

The current work found that miR-146a can be accurate in the diagnosis of patients with RA with an AUC of 0.836 at a cut-off value of ≥1.433 folds with a sensitivity and specificity of 82.5% and 100%, respectively. Therefore, it can distinguish patients from healthy controls. Different studies documented elevated expression levels of miR-146a in different sample types, such as synovial fluid, tissues, and fibroblasts, signifying its role as a potential biomarker for RA diagnosis [55,56,57]. This study used the least invasive procedure to detect the expression levels, as it can be applied to a patient’s diagnosis as well as follow-up. Regarding the correlation between the level of miR-146a expression and the disease course, we demonstrated that there was no correlation between the expression levels and DAS28, ESR, and RF. Similarly, Li et al. did not find an association between miR-146a expression levels and DAI in both peripheral blood and synovial fluid [58].

Regarding the OA patients, no association was detected between miR-146a and different disease severity scales such as total KOOS score, chair stand test, stair climb test, and Kellgrn–Lawrence grading radiological scale. The small number of participants included in the study was considered a limitation. However, our results, which highlighted the value of lnc-PVT1 and miR-146a as biomarkers for RA and OA, could be a beginning for more studies on a wider scale to understand the exact role of these biomarkers in joint disorders and the ability to use them as a therapeutic target.

## 5. Conclusions

In conclusion, our study demonstrated that lnc-PVT1 and miR-146a could be considered as promising biomarkers for the diagnosis of RA and OA and may have an essential role as therapeutic targets in the future. However, no association was detected between both biomarkers and the pathogenesis of RA and OA. Further studies are required to confirm the current observations and to highlight the possibility of using them as therapeutic targets in RA and OA. However, the current study has a limitation due to the small number of participating subjects.

## Figures and Tables

**Table 1:** Comparisons of demographic characteristics in different study groups. The table illustrates that there was a statistically significant difference with a *p*-value < 0.05 between the study groups as regards age, with a lower mean age among the RA group. On the other hand, there was no statistically significant difference with *p*-value > 0.05 as regards sex distribution among groups.

Variables	OA (*n* = 40)	RA (*n* = 40)	HCs (*n* = 40)	*p*-Value
	Mean ± SD	Mean ± SD	Mean ± SD	
Age (year)	53.5 ± 6.7	38.4 ± 10.1	52.8 ± 8.4	<0.001 *
Sex				
Male	10 (25%)	5 (12.5%)	7 (17.5%)	0.4
Female	30 (75%)	35 (87.5%)	33 (82.5%)	

(*) indicates that significant difference is present.

**Table 2:** Comparisons of laboratory investigations in different study groups. The table illustrates that there was a statistically significant difference with *p*-value < 0.05 between the study groups as regards creatinine, urea, ALT, ESR, and HB levels, with a lower mean of creatinine, urea, and ALT among the OA group, and a higher mean of ESR and a lower mean of HB among the RA group. On the other hand, there was no statistically significant difference with a *p*-value > 0.05 as regards other investigations.

Variables	OA (n = 40)		RA (n = 40)		HCs (n = 40)		*p*-Value
	Mean	SD	Mean	SD	Mean	SD	
Creatinin	0.73	0.32	0.82	0.22	0.91	0.30	0.02 *
Urea	22.4	4.4	---	---	25.3	5.4	0.01 *
AST	17.7	6.4	---	---	15.2	6.8	0.09
ALT	21.4	4.4	25.9	11.1	22.1	9.2	0.05 *
ESR	27.1	22.8	38.2	22.6	25.6	18.3	0.02 *
HB	13.3	1.5	11.9	1.6	12.3	1.2	<0.001 *
WBC	7.3	2.8	7.8	2.5	6.5	1.6	0.06
PLT	250.9	53.6	281.4	83.2	254.7	66.2	0.1

AST: aspartate transaminase, HB: haemoglobin, ALT: alanine aminotransferase, WBC: white blood cells, ESR: erythrocyte sedimentation rate, PLT: platelets. (*) indicates that significant difference is present.

**Table 3:** Comparisons of markers in different study groups. The table illustrates that there was a statistically significant difference with *p*-value < 0.05 between study groups as regards the lnc-PVT1 marker, with a higher fold among the RA group and a lower fold among the OA group. In addition, there was a statistically significant higher miR146a marker fold change with a *p*-value < 0.05 between both the RA and OA groups and HCs but no difference between the OA and RA groups.

Variables	OA (*n* = 40)		RA (*n* = 40)		Control (*n* = 40)		*p*-Value
	Median	IQR	Median	IQR	Median	IQR	
lnc-PVT1	0.22	0.23	2.62	10.1	1	0.2	<0.001 ^a,c^
							0.01 ^b^
miR-146a	4.6	1.9	6.9	10.6	1	0.2	<0.001 ^a,b^
							0.06 ^c^

^a^: significant difference between OS and control, ^b^: significant difference between RA and control, ^c^: significant difference between OS and RA.

**Table 4:** Description of disease criteria for activity and severity among RA group.

Criteria for Activity and Severity	Mean	SD	Range
TJC	5.3	5.4	0–25
SJC	5.5	5.6	0–25
DAS28 score	3	1.7	0.5–7.3
		Number	%
Deformity	No	9	22.5%
	Yes	31	77.5%
Arthritis	No	12	30%
	Yes	28	70%
RF	Negative	11	27.5%
	Positive	29	72.5%
ANA	Negative	34	85%
	Positive	6	15%
Activity	Remission	18	45%
	Low	4	10%
	Moderate	13	32.5%
	High	5	12.5%
Plain X-ray	Erosion deformity	7	17.5%
	Owing osteopenia	33	82.5%

TJC: tender joint count, SJC: swollen joint count, DAS28: disease activity score, RF: rheumatoid factor, ANA: antinuclear antibodies.

**Table 5:** Correlation between lnc-PVT1 and miR-146a markers with disease characters among RA group. The table illustrates that there is a statistically significant positive correlation with a *p*-value < 0.05 between the lnc-PVT1 marker and SJC among the RA group. There was no correlation with other study variables. On the other hand, there is no statistically significant correlation (*p*-value > 0.05) between miR146a and other variables among the RA group.

Variables	lnc-PVT1		miR-146a	
	r	*p*-Value	r	*p*-Value
Disease duration	0.21	0.2	−0.15	0.4
TJC	0.08	0.6	−0.13	0.4
SJC	0.35	0.03 *	−0.10	0.5
DAS28 score	0.16	0.3	−0.19	0.2

TJC: tender joint count, SJC: swollen joint count, DAS28: disease activity score.

**Table 6:** Sensitivity and specificity of lnc-PVT1 and miR146a markers in diagnosis of RA diseases. Sensitivity and specificity test for lnc-PVT1 marker in diagnosis of RA disease was 62.5% and 90% at cut off 1.15. For miR146a marker the sensitivity of RA diagnosis was 82.5% and 100% specificity at cut off 1.433.

Variable	Sensitivity	Specificity	AUC	CI	Cut Off Point (*p*-Value)
RA					
lnc-PVT1	62.5%	90%	65.4%	0.51–0.79	1.15 (*p* = 0.01)
miR 146a	82.5%	100%	83.6%	0.72–0.95	1.433 (*p* = 0.001)

AUC: area under curve, CI: confidence interval.

**Table 7:** Correlation between PVT1 and miR-146a markers with demographic and laboratory investigations among RA group. The table describes the relationship between both biomarkers and the biochemical characteristics of RA patients. They showed no correlation between both and any of the biochemical and demographic characteristics.

Variables	PVT1		miR-146a	
	r	*p*-Value	r	*p*-Value
Age (year)	0.10	0.5	−0.04	0.9
BMI (kg/m^2^)	0.16	0.3	−0.18	0.3
Laboratory investigations				
Creatinine	−0.06	0.7	−0.19	0.3
ALT	−0.10	0.5	−0.28	0.08
ESR	−0.02	0.9	0.03	0.8
HB	0.006	0.9	0.04	0.8
WBC	0.2	0.2	0.19	0.2
PLT	0.09	0.6	−0.003	0.9

**Table 8:** Description of disease severity scales among OA group.

Disease Severity Scales		Mean	SD	Range
Total koos score		47.3	13.4	23–66
Chair stand test		11.4	6.9	5–30
Stair climb test		19.7	8	11.8–43
Kellgrn–Lawrence Grading radiological Scale	Stage 2	15	37.5%	
	Stage 3	17	42.5%	
	Stage 4	8	20%	

Koos score: The Knee injury and Osteoarthritis Outcome Score.

**Table 9:** Sensitivity and specificity of lnc-PVT1 and miR-146a markers in diagnosis of OA. Sensitivity and specificity tests for the lnc-PVT1 marker in the diagnosis of OA disease were (100%) and (77.5%) at cut off (0.835). For the miR-146a marker, the sensitivity of OA diagnosis was 90% and the specificity was 100% at the cut off (2.05).

Variable	Sensitivity	Specificity	AUC	CI	Cut Off Point (*p*-Value)
**OA**					
lnc-PVT1	100%	77.5%	98.9%	0–1	0.865 (*p* = 0.001)
miR-146a	90%	100%	90%	0.81–0.99	2.05 (*p* = 0.001)

AUC: area under curve, CI: confidence interval.

**Table 10:** Correlation between PVT1 and miR-146a markers with demographic and laboratory investigations among OA group. The table shows the correlation between both biomarkers and the biochemical characteristics of OA patients. There was no correlation found between either biomarker and any of the biochemical or demographic characteristics.

Variables	PVT1		miR 146a	
	r	*p*-Value	r	*p*-Value
Age (year)	−0.04	0.8	−0.15	0.4
BMI (kg/m^2^)	0.23	0.1	−0.04	0.8
Laboratory investigations				
Creatinin	0.13	0.4	0.19	0.2
Urea	−0.27	0.09	0.04	0.8
AST	−0.13	0.4	0.07	0.7
ALT	−0.19	0.3	−0.09	0.6
ESR	0.18	0.3	0.19	0.2
HB	0.07	0.7	0.13	0.4
WBC	−0.11	0.5	−0.23	0.1
PLT	−0.23	0.2	−0.01	0.9

## Data Availability

Not applicable.

## Supplementary Materials

The following are available online at https://www.mdpi.com/article/10.3390/life11121382/s1, Figure S1: A schematic description of the study outline for the participant groups. (a) refers to a recapitulative figure describing the clinical examination, laboratory investigations, and biomarkers investigation carried out on the RA group, (b). refers to a recapitulative figure describing the clinical examination, laboratory investigations, and biomarkers investigation carried out on the OA group.
