# Supplementary figures and images for: Long Non-Coding RNA PVT1 and Its Target miRNA-146a as Potential Prognostic Biomarkers in Rheumatoid Arthritis Patients
Source: Life (Basel). 2021 Dec 10;11(12):1382. doi: 10.3390/life11121382 (PMC8706643; doi:10.3390/life11121382)

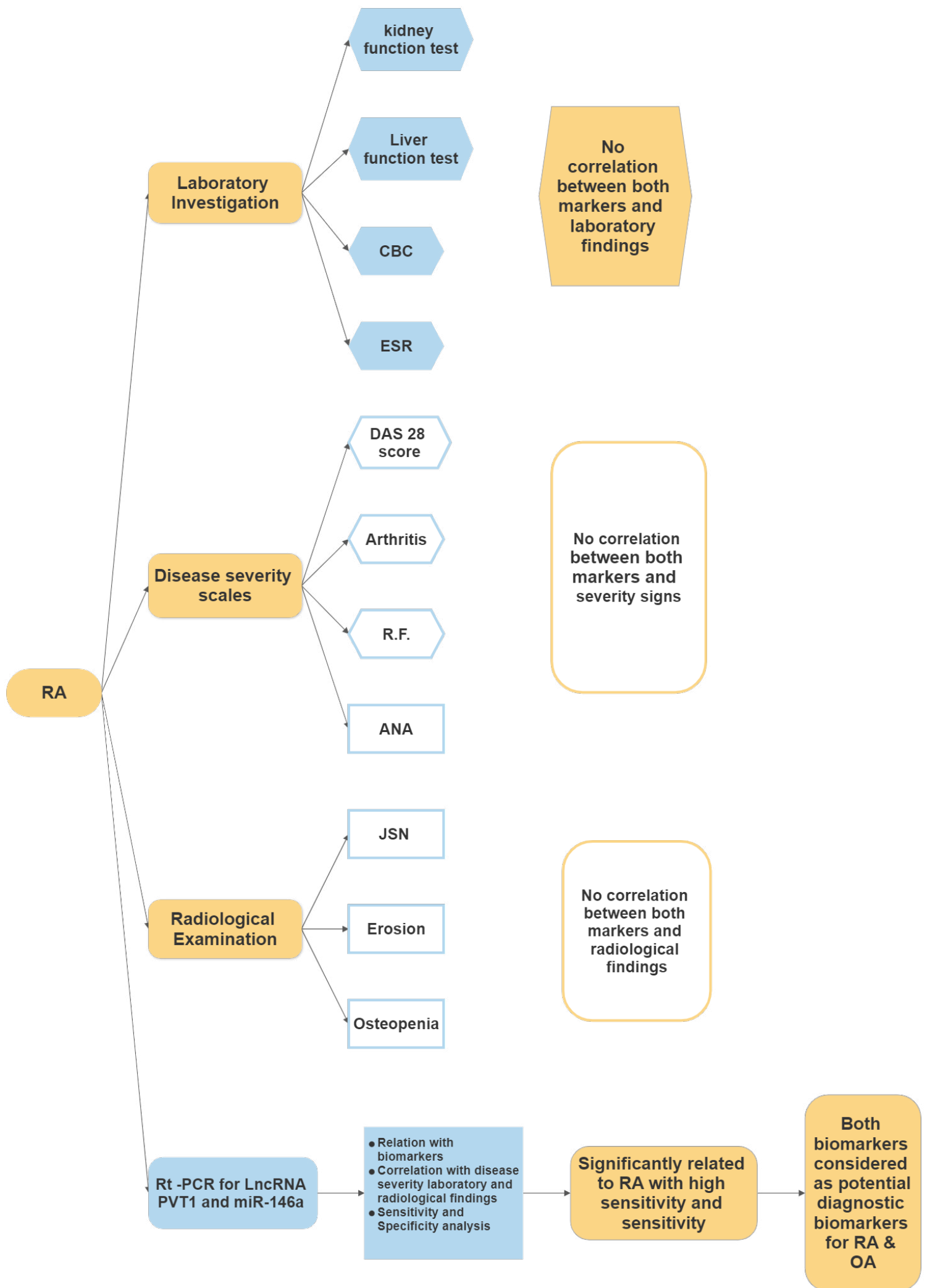

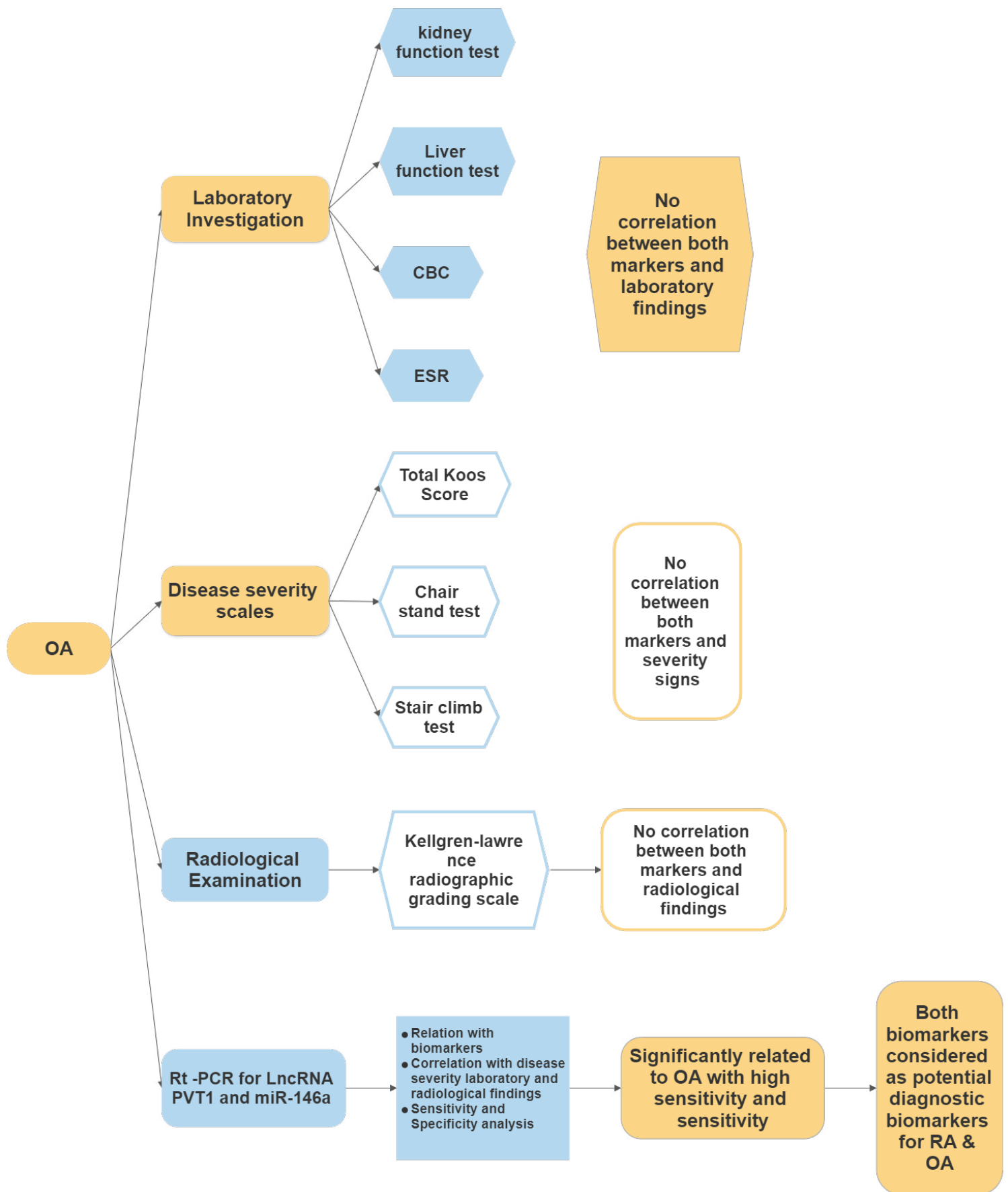

Supplement: Supplementary file 1 [file life-11-01382-s001.zip › life-1455298-supplementary.pdf]
